# Gastrointestinal Parasitic Infections and Immunological Status of HIV/AIDS Coinfected Individuals in Nigeria

**DOI:** 10.5334/aogh.2554

**Published:** 2019-07-05

**Authors:** Emmanuel Ochigbo Udeh, R. N. N. Obiezue, F. C. Okafor, C. B. Ikele, I. C. Okoye, Chidiebere A. Otuu

**Affiliations:** 1 Department of Zoology and Environmental Biology, Faculty of Biological Sciences, University of Nigeria, Nsukka, NG; 2 HIV/AIDS Care and Treatment Unit, Centre for Integrated Health Programs, Benue State Regional Office, Makurdi, NG

## Abstract

**Background:**

Parasitic infections of the gastrointestinal tract is one of the highest causes of morbidity and mortality among HIV infected individuals. This is due to the colonization of the intestinal tract by parasites influenced by induced enteropathy caused by HIV infection. CD^+^4 t-lymphocytes count is a marker of the immune status of HIV infected individuals.

**Objective:**

This study investigated the prevalence of gastrointestinal parasitic infections among HIV coinfected individuals in relation to their immunological status.

**Methods:**

CD^+^4 t-lymphocytes count was determined using fluorescence-activated cell sorting (FACS) count system. Parasitological examination of faecal samples was conducted using direct wet mount, modified Z-N and Giemsa stain techniques. All prepared slides were examined under x10 and x40 objectives.

**Findings:**

Out of the 891 HIV seropositive participants on antiretroviral therapy that were studied, 641 (71.9%) had CD^+^4 counts equals to or greater than 500 cells/mm^3^. All other seropositive participants had CD^+^4 counts below 500 cells/mm^3^. Gastrointestinal parasitic infections were recorded in 187 (20.9%) seropositive participants, with females (n = 108, 12.1%) having more infections than males. Multiple gastrointestinal parasitic infections were recorded in 28 (3.1%) seropositive participants. Out of the 150 seronegative participants, 79 (52.7%) of them had at least one gastrointestinal parasitic infection. Female seronegative participants also accounted for higher infection rate (n = 42, 28.0%) than males (n = 37, 24.7%). Multiple infections were also recorded in 18 (12.0%) seronegative individuals. The overall prevalence rate of infection between both positive and negative individuals was 25.5%. There was statistical significant difference in the infections of *Cryptosporidium parvum* (p < 0.003), *Cyclospora cayetanensis* (p < 0.011) and *Cystoisospora belli* (p < 0.011) between HIV seropositive and HIV seronegative individuals. Also, there was statistical significant difference in the infections of hook worm (p < 0.002) and *Trichuris trichiura* (p < 0.020) between seronegative and seropositive individuals. Gastrointestinal parasitic infection rate was significantly higher among seropositive participants with CD^+^4 counts between 200 and 350 cells/mm^3^ (n = 109, 58.3%).

**Conclusion:**

The study shows that HIV infected individuals continue to experience gastrointestinal infections even with antiretroviral treatment, especially those with CD^+^4 counts below 350 cells/mm^3^. Health care providers should prioritise routine screening of HIV patients for gastrointestinal parasites and provide prompt treatment. Antiparasitic drugs should also be provided as prophylaxis.

## Introduction

Gastrointestinal parasitic infections continue to cause high morbidity and mortality among immunocompromised individuals, especially those with HIV/AIDS. The effect of the HIV virus on the helper t-cell monocytes, macrophages, and neutrophils result in the increased susceptibility of infected individuals to multiple gastrointestinal parasitic infections. The colonization of the intestinal tract by parasites is influenced by induced enteropathy as a result of HIV infection [1,2,3]. The gut of HIV infected individuals may not be a favourable environment for the establishment and survival of extracellular parasites, however intracellular and mucosal dwelling parasites may not be adversely affected by the pathologic changes [1,2,4]. The frequency of infection of intracellular intestinal protozoans like *Cryptosporidium parvum, Cyclospora cayetanensis*, and *Cystoisospora belli* have been associated with increasing establishment, and survival results in higher prevalence in HIV infected individuals with enteropathy when compared with persons not affected by the virus.

In most countries of sub-Saharan Africa, including Nigeria where there is limited access to viral load quantification, HIV patients are routinely monitored for immunological levels by enumerating their CD^+^4 t-lymphocytes count. CD^+^4 count is also used as a measure of the presence or otherwise of opportunistic infections and failure or success of treatment. Patients on highly active antiretroviral therapy (HAART) are expected to achieve an increase in CD^+^4 cells and recovery of the immune functions. Treatment is considered successful if there is a rise in CD^+^4 t-lymphocytes count. Treatment failure, on the other hand, is indicated by a 30% fall in CD^+^4 t-lymphocytes count [5,6,7,8].

Since CD^+^4 t-lymphocytes level of HIV infected individual is also a determinant for the presence or absence of opportunistic infections, especially gastrointestinal parasitic infections, which accounts for high rates of morbidity and mortality among coinfected individuals, the objective of this study was to determine the prevalence of gastrointestinal parasitic infections among HIV coinfected individuals in relation to their immunological status.

## Materials and Methods

### Study Area

Makurdi is the state capital of Benue State, in North Central Nigeria. It is located along the River Benue on latitude 7.74°N and longitude 8.51°E. It is the most urbanised city in the state with a population of about 292,645 inhabitants who are mainly civil servants, traders, students, and artisans. It has one of the highest burden of HIV/AIDS in the state with a prevalence rate of 8.0% [9]. The study was conducted at the HIV treatment center of General Hospital Makurdi.

### Study Population

The study population were HIV clients registered for care and treatment at the center. A total of 3,772 clients were active on treatment as at the time of commencement of the six months study in June, 2018. A total of 891 HIV clients on treatment and 150 HIV seronegative individuals participated in the study.

### Inclusion and Exclusion Criteria

All HIV/AIDS clients 15 years and above who were active on antiretroviral treatment (ART) for a minimum period of 12 months were included in the study. All clients below the age of 15 years and those less than 12 months on ART were excluded from the study.

### Ethical approvals and permissions

Ethical approval for the study was granted by the ethical review committee of the Benue State Ministry of Health and Human Services. Also, a letter of approval to have access to the health facility was granted by the Benue State Hospitals Management Board. The informed consent of each client that participated in the study was also obtained.

### Study Designs

A hospital based case-control study was carried out at the HIV treatment center of General Hospital Makurdi. Clients’ clinical case notes were reviewed and information were abstracted. Retrospective information obtained included date of confirmation of HIV seropositive status, WHO clinical stage of HIV disease at enrolment into treatment, baseline CD^+^4 counts, viral load assessment while on ART, history of opportunistic infections (OIs) and ART regimens at start of treatment and follow ups. Also, apparently healthy HIV seronegative individuals who have confirmed HIV seronegative status were recruited into the study from the HIV counselling and testing unit of the center to serve as control.

### CD^+^4 T-lymphocytes Enumeration

From each participant, 3 ml of blood was collected into an ethylenediaminetetraacetic acid (EDTA) anti-coagulated tube for CD^+^4 t-cell count. The fluorescence-activated cell sorting (FACS) count (Becton Dickinson immunocytometry system, Singapore) was used for the immunophenotyping of lymphocytes. CD^+^4 reagent tubes were vortexed and opened with the coring station and 50 µl of whole blood from EDTA tube added. These were vortexed and incubated for one hour in the dark at room temperature. The tubes were uncapped and 50 µl of fixative added. The tubes were recapped and vortexed for five seconds while standing upright before subjecting it to the FACS Count instrument for the immunophenotyping of lymphocytes.

### Parasitological Examination of Faecal Samples

Each of the participants was provided with two well labelled sterile screw-capped containers to provide stool samples which were collected in the morning, first on the day prior to their clinic visit and the second on the day of their scheduled clinic visit. On arrival in the laboratory, direct wet mount of the stool samples in normal saline (0.85% NaCl) were prepared and examined under a light microscope (×10 and ×40 objectives) for the presence of vegetative forms, larvae, and ova of helminths. All stool samples were processed using mini Parasep^R^ SF faecal parasite concentrator (manufactured by Apacor Ltd, Wokingham, England. Product Code 108920). Each prefilled mini Parasep^R^ SF faecal parasite concentrator contained 3.3 ml of sodium acetate-acetic acid-formalin solution (SAF) and triton X solution. Taking one parasep concentrator and using the spoon on the end of the filter, a scoop of faecal sample was taken from each study sample and introduced into the sample chamber of the concentrator containing the fixative. This was mixed thoroughly using the spoon. Hard samples were broken using the spoon. The parasep concentrator was then sealed by screwing in the filter and the sedimentation cone unit. This was then emulsified by vortexing, with the sedimentation cone pointing upwards. After emulsification, the parasep concentrator was inverted into a centrifuge and centrifuged for two minutes. The concentrator was then taken out, opened and the supernatant discarded. Three slides were prepared from each of the concentrated samples by pipetting one drop of the sediment onto three grease free slides. A drop of Lugo’s iodine was added onto one of the slides and covered with a cover slip for the identification of intestinal flagellates and amoebae. For the identification of coccidians, the second slides were fixed in 70% methanol for two minutes, air dried and stained with Z-N carbol fuchsin for ten minutes. This preparation was gently rinsed in slow running tap water, decolourised with 1% acid alcohol for one minute, rinsed in water again and then stained with 1% methyl blue for 1 minute and finally rinsed again in water and air dried. The third slides were immersed in diluted stain of Giemsa, rinsed gently in buffer water and air dried. All the slides were examined under a light microscope using first ×10 objective and then ×40 objective.

### Statistical Analysis

The results of the study were analysed using chi square test and SPSS version 22 and summarized using frequency tables.

## Results

A total of 891 HIV seropositive clients on antiretroviral therapy and 150 apparently healthy HIV seronegative individuals participated in the study. Males constitute 50.3% (n = 448) of the seropositive group while female constitutes 51.3% (n = 77) of the seronegative group (Table 1).

**Table 1:** Age and sex distribution of study participants.

	HIV seropositive participants (Case)			HIV seronegative participants (control)		

Age (years)	Male	Female	Total (%)	Male	Female	Total (%)

15–24	53	54	107 (12.0%)	16	9	25 (16.7%)
25–34	114	171	285 (32.0%)	23	24	47 (31.3%)
35–44	117	129	246 (27.6%)	25	29	54 (36.0%)
45–54	120	61	181 (20.3%)	6	8	14 (9.3%)
>55	44	28	72 (8.1%)	3	7	10 (6.7%)
**Total**	**448 (50.3%)**	**443 (49.7%)**	**891 (100%)**	**73 (48.7%)**	**77 (51.3%)**	**50 (100%)**

Immunological analysis of the HIV seropositive participants show that 71.9% (n = 641) had CD^+^4 counts equals to or greater than 500 cells/mm^3^. All other seropositive participants had CD^+^4 counts below 500 cells/mm^3^; with 133 (14.9%), 114 (12.8%) and 3 (0.3%) having 300 to less than 500, 200 to less than 350, and less than 200 cells/mm^3^ of t-lymphocytes counts respectively (Table 2).

**Table 2:** Distribution of HIV positive participants based on their CD^+^4 t-lymphocytes levels.

CD^+^4 Levels (cells/mm^3^)	No. of participants (%)	p-value	

<200	3 (0.3%)	0.001	*
≥200 <350	114 (12.8%)		
≥350 <500	133 (14.9%)		
≥500	641 (71.9%)		
**Total**	**891 (100.0%)**		

A total of 187 (20.9%) seropositive individuals had at least one gastrointestinal parasitic infection. Female seropositive individuals (n = 108, 12.1%) had more infections than males (n = 79, 8.8%). Multiple infections were recorded in 28 seropositive individuals (3.1%). A total of 79 (52.7%) HIV seronegative individuals had at least one gastrointestinal parasitic infection. Female seronegative individuals also accounts for higher infection rate (n = 42, 28.0%) than males (n = 37, 24.7%). Multiple infections were also recorded in 18 (12.0%) seronegative individuals (Table 3). The overall prevalence rate of infection between both positive and negative individuals was 25.5%.

**Table 3:** Distribution of gastrointestinal parasites among HIV seropositive and seronegative individuals.

Gastrointestinal Parasites	HIV positive			HIV Negative			p-value	

	Male	Female	Total	Male	Female	Total		

*Blastocystis hominis*	2(1.1%)	4(2.1%)	6(2.3%)	1(1.3%)	0(0.0%)	1(0.4%)	0.333	
*Cryptosporidium parvum*	8(4.3%)	11(5.9%)	19(7.1%)	0(0.0%)	0(0.0%)	0(0.0%)	0.003	*
*Cyclospora cayetanensis*	6(3.2%)	3(1.6%)	9(3.4%)	0(0.0%)	0(0.0%)	0(0.0%)	0.011	*
*Cystoisospora belli*	3(1.6%)	6(3.2%)	9(3.4%)	0(0.0%)	0(0.0%)	0(0.0%)	0.011	*
*Enterocytozoon bieneusi*	2(1.1%)	3(1.6%)	5(1.9%)	0(0.0%)	0(0.0%)	0(0.0%)	0.059	
*Entamoeba histolytica*	8(4.3%)	11(5.9%)	19(7.1%)	3(3.8%)	5(6.3%)	8(3.0%)	0.993	
*Entamoeba coli*	8(4.3%)	13(7.0%)	21(7.9%)	2(2.5%)	3(3.8%)	5(1.9%)	0.202	
*Giardia lamblia*	8(4.3%)	12(6.4%)	20(7.5%)	2(2.5%)	5(6.3%)	7(2.6%)	0.647	
*Balantidium coli*	4(2.1%)	6(3.2%)	10(3.8%)	2(2.5%)	2(2.5%)	4(1.5%)	0.924	
*Ascaris lumbricoides*	2(1.1%)	2(1.1%)	4(1.5%)	1(1.3%)	0(0.0%)	1(0.4%)	0.619	
*Taenia sp*.	14(7.5%)	10(5.3%)	24(9.0%)	8(10.1%)	7(8.9%)	15(5.6%)	0.204	
*Hookworm*	2(1.1%)	3(1.6%)	5(1.9%)	5(6.3%)	5(6.3%)	10(3.8%)	0.002	*
*Strongyloides stercoralis*	3(1.6%)	2(1.1%)	5(1.9%)	2(2.5%)	2(2.5%)	4(1.5%)	0.342	
*Trichuris trichiura*	1(0.5%)	2(1.1%)	3(1.1%)	4(5.1%)	2(2.5%)	6(2.3%)	0.020	*
*E. histolytica* & *Taenia sp*.	3(1.6%)	7(3.7%)	10(3.8%)	2(2.5%)	3(3.8%)	5(1.9%)	0.754	
*E. bieneusi* & *C. belli*	(0.0%)	1(0.5%)	1(0.4%)	(0.0%)	0(0.0%)	0(0.0%)	0.401	
*E. coli* & *G. lamblia*	1(0.5%)	4(2.1%)	5(1.9%)	2(2.5%)	3(3.8%)	5(1.9%)	0.170	
*E. histolytica* & *G. lamblia*	4(2.1%)	5(2.7%)	9(4.6%)	3(3.8%)	3(3.8%)	6(2.3%)	0.381	
*C. belli* & *S. stercoralis*	(0.0%)	3(1.6%)	3(1.1%)	0(0.0%)	2(2.5%)	2(0.8%)	0.620	
Total	79(8.8%)	108(12.1%)	187(20.9%)	37(24.7%)	42(28.0%)	79(52.7%)		

There was statistical significant difference in the infections of *Cryptosporidium parvum* (p < 0.003)*, Cyclospora cayetanensis* (p < 0.011) *and Cystoisospora belli* (p < 0.011) between HIV seropositive and HIV seronegative individuals. Also, there was statistical significant difference in the infections of hook worm (p < 0.002) and *Trichuris trichiura* (p < 0.020) between seronegative and seropositive individuals (Table 3).

Gastrointestinal parasitic infection rate was higher among individuals with CD^+^4 counts between 200 and 350 cells/mm^3^ (n = 109, 58.3%) (Table 4). This was seen to be significant among females (n = 64, 34.2%) and males (n = 45, 24.1%). Also, 66 individuals (35.3%) with CD^+^4 counts between 350 and less than 500 cells/mm^3^ were infected with gastrointestinal parasites. Females (n = 36, 19.3%) also accounts for higher rate than males (n = 30, 16.0%) (Table 5).

**Table 4:** Distribution of gastrointestinal parasite in relation to CD^+^4 cell counts.

Parasites	CD^+^4 counts (cells/mm^3^)				Total

	<200	≥200 <350	≥350 <500	≥500	

*Blastocystis hominis*	2	4	0	0	6
*Cryptosporidium parvum*	1	11	7	0	19
*Cyclospora cayetanensis*	0	6	3	0	9
*Cystoisospora belli*	0	5	4	0	9
*Enterocytozoon bieneusi*		3	2	0	5
*Entamoeba histolytica*		10	7	2	19
*Entamoeba coli*		14	7	0	21
*Giardia lamblia*		9	9	2	20
*Balantidium coli*		6	4		10
*Ascaris lumbricoides*		3	1		4
*Taenia sp.*		10	9	5	24
*Hookworm*		3	2		5
*Strongyloides stercoralis*		4	1		5
*Trichuris trichiura*		3			3
*E. histolytica* & *Taenia sp*.		6	4		10
*E. bieneusi* & *C. belli*		1			1
*E. coli* & *G. lamblia*		4	1		5
*E. histolytica* & *G. lamblia*		5	4		9
*C. belli* & *S. stercoralis*		2	1		3
**Total**	3	109	66	9	187

**Table 5:** Distribution of gastrointestinal parasites in relation to CD^+^4 cell counts and gender.

Parasite	Male				Female				Total

	<200	≥200 <350	≥350 <500	≥500	<200	≥200 <350	≥350 <500	≥500	

*Blastocystis hominis*	1	1			1	3			6
*Cryptosporidium parvum*		5	3		1	6	4		19
*Cyclospora cayetanensis*		3	3			3			9
*Cystoisospora belli*		2	1			3	3		9
*Enterocytozoon bieneusi*		1	1			2	1		5
*Entamoeba histolytica*		5	3			5	4	2	19
*Entamoeba coli*		6	2			8	5		21
*Giardia lamblia*		3	4	1		6	5	1	20
*Balantidium coli*		2	2			4	2		10
*Ascaris lumbricoides*		1	1			2			4
*Taenia sp.*		7	5	2		3	4	3	24
*Hookworm*		1	1			2	1		5
*Strongyloides stercoralis*		2	1			2			5
*Trichuris trichiura*		1				2			3
*E. histolytica* & *Taenia sp*.		2	1			4	3		10
*E. bieneusi* & *C. belli*						1			1
*E. coli* & *G.lamblia*		1				3	1		5
*E. histolytica* & *G. lamblia*		2	2			3	2		9
*C. belli* & *S. stercoralis*						2	1		3
**Total**	1	45	30	3	2	64	36	6	187

## Discussions

The use of CD^+^4 t-lymphocytes count to monitor HIV patients on ART has been shown to be highly indicative of their morbidity status. It has been established that a weakened immune system, depicted by a low CD^+^4 count, results in high susceptibility of the individuals to comorbidities. This has been documented in HIV infected individuals with low CD^+^4 counts [10,11,12]. This is why the World Health Organization (WHO) recommends the use of CD^+^4 count to monitor opportunistic infections [5].

The findings of this study have shown that a significant proportion of HIV infected individuals on antiretroviral therapy still experience low CD^+^4 t-lymphocytes count and high intestinal parasitic comorbidities. The low CD^+^4 t-lymphocytes count may be as a result of poor adherence to treatment regimens [13,14,15,16]. Low CD^+^4 counts and symptomatic HIV disease have been associated with treatment failure [17].

Out of the total 891 HIV clients on antiretroviral therapy that were studied, 250 (28%) of them have low CD^+^4 counts below 500 cells/mm^3^, with a significant proportion of 117 (13.1%), having a CD^+^4 count below 350 cells/mm^3^. Gastrointestinal parasitic infection was high among individuals who had CD^+^4 count less than 500 cells/mm^3^ and significantly high among those with CD^+^4 count less than 350 cells/mm^3^. Only nine individuals with CD^+^4 count greater than 500 cells/mm^3^ were seen with monoparasitic infections. Multiple gastrointestinal parasitic infections where very common among those with CD^+^4 count below 350 cells/mm^3^. The study also shows that *Taenia sp., Entamoeba coli, Giardia lamblia, Entamoeba histolytica, Cryptosporidium parvum, Balantidium coli, Cyclospora cayetanensis, Cystoisospora belli, Blastocystis hominis, Enterocytozoon bieneusi*, hook worm, *Strongyloides stercoralis, Ascaris lumbricoides*, and *Trichuris trichiura* were isolated from individuals with CD^+^4 count below 500 cells/mm^3^. However, only single infections of *Entamoeba histolytica* in two individuals, *Giardia lamblia* in two individuals and *Taenia sp*. in five individuals were isolated from those with CD^+^4 count above 500 cells/mm^3^. The study also shows that multiple gastrointestinal parasitic infections occur only in individuals with low CD^+^4 counts less than 500 cells/mm^3^ with significant proportion among those with CD^+^4 counts less than 350 cells/mm^3^. Dual infections of *E. histolytica* and *Taenia sp*. were recorded in ten individuals while *E. histolytica* and *G. lamblia* were recorded in nine individuals. Also, dual gastrointestinal parasitic infections of *E. coli* and *G. lamblia* were recorded in five individuals, while *C. belli* and *S. stercoralis* were recorded in three individuals. One individual with CD^+^4 count between 200 and less than 350 cells/mm^3^ had a dual infection of *E. bieneusi* and *C. belli*. Three individuals with CD^+^4 count less than 200 cells/mm^3^ were infected with *B. hominis* (n = 3) and *C. parvum* (n = 1). This study is in corroboration with similar findings already documented in similar settings [18,19,20,21,22,23,24,25,26,27]. In a related study, a 13.3% prevalence of intestinal parasitic infections was recorded among HIV infected individuals with significant high rate of microsporidians and coccidians associated with CD^+^4 counts below 200 cells/mm^3^ [28]. Also, a high intestinal parasitic infection rate of 15.3% among HIV infected individuals with significant infections among those with CD^+^4 counts below 200 cells/mm^3^ had been established [29].

The study results show that parasitic infections of the gastrointestinal tract remains a burden among HIV infected individuals even while on antiretroviral therapy. Patients with low CD^+^4 counts, especially below 350 cells/mm^3^ are significantly associated with increased rate of gastrointestinal infections.

In conclusion, periodic screening of HIV infected individuals for gastrointestinal parasitic infections should be carried out at least every six months and especially during every CD^+^4 t-lymphocytes count evaluation. This will reduce morbidity, enhance antiretroviral treatment success and ultimately improve their well-being. The inclusion of antiparasitic drugs, even as prophylaxis, among the routine drug regimens for care and treatment of infected persons is highly recommended.
